# Serum Levels of Insulin-like Growth Factor I, II, and Binding Protein 3, Transforming Growth Factor *β*-1, Soluble Fas Ligand and Superoxide Dismutase Activity in Stomach Cancer Cases and Their Controls in the JACC Study

**DOI:** 10.2188/jea.15.S120

**Published:** 2005-08-18

**Authors:** Hiroshi Yatsuya, Hideaki Toyoshima, Koji Tamakoshi, Akiko Tamakoshi, Takaaki Kondo, Norihiko Hayakawa, Kiyomi Sakata, Shogo Kikuchi, Yoshiharu Hoshiyama, Yoshihisa Fujino, Tetsuya Mizoue, Noritaka Tokui, Takesumi Yoshimura

**Affiliations:** 1 Department of Public Health/ Health Information Dynamics, Nagoya University Graduate School of Medicine.; 2 Department of Preventive Medicine/ Biostatistics and Medical Decision Making, Nagoya University Graduate School of Medicine.; 3 Department of Medical Technology, Nagoya University School of Health Sciences.; 4 Department of Epidemiology, Hiroshima University Research Institute for Radiation Biology and Medicine.; 5 Department of Public Health, Wakayama Medical University.; 6 Department of Public Health, Aichi Medical University.; 7 Department of Public Health, Showa University School of Medicine.; 8 Fukuoka Institute of Occupational Health.; 9 Department of Clinical Epidemiology, Institute of Industrial Ecological Sciences, University of Occupational and Environmental Health.; 10 Department of Preventive Medicine, Graduate School of Medical Sciences, Kyushu University.; 11 Fukuoka Institute of Health and Environmental Sciences.; 12 See acknowledgment for the investigators (name and affiliation) involved in the JACC Study

**Keywords:** IGFs, TGF *β*1, sFas, SOD, Stomach Neoplasms, Helicobacter pylori

## Abstract

BACKGROUND: The prognosis of stomach cancer with advanced stage remains poor. New biomarkers of the disease that may contribute to establish the potential screening strategy would be of value for the early detection of individuals at high risk of the disease.

METHODS: We conducted a prospective, nested case-control analysis among apparently healthy men and women who were followed for up to 8 years in the Japan Collaborative Cohort (JACC) Study, to evaluate serum levels of insulin-like growth factor I, II, and binding protein 3 (IFG-I, IGF-II, and IGFBP-3), transforming growth factor *β*-1 (TGF *β*1), soluble fas (sFas) and superoxide dismutase activity (SOD) in 210 stomach cancer cases diagnosed in the JACC Study in relation to those levels in their 410 controls.

RESULTS: Among 6 serum biomarkers tested for case-control differences, only sFas level in female stomach cancer cases was significantly higher than that of controls (2.22 pg/ml vs. 2.04 pg/mL, respectively; P = 0.013 by two-way analysis of covariance controlling for matching variable).

CONCLUSION: None of the biomarkers consistently predicted future risk of stomach cancer in both men and women in the present analysis. Serum sFas level in women, however, should be studied much more thoroughly whether it provides meaningful refinement of risk stratification, or it elucidate the mechanisms of tumorigenesis in women.

Gastric cancer is the second most common cancer worldwide, with about 800,000 new cases diagnosed every year. ^1^ Steady declines in the incidence rates have been observed worldwide in the last few decades. Although the exact causes of the decline are not well understood, they may include improvements in diet and food storage and a decline in the prevalence of *Helicobacter pylori* infection.^2^

Molecular mechanisms of the development and progression of gastric cancer such as the role of E-cadherin, transforming growth factor *β* or RUNX3 have also been extensively studied by the integrated pathological research, and these molecular technologies may promise to prevent and treat the disease in future.^3^^-^^5^ Advances in diagnosis and treatment have resulted in the improvement in long-term survival for patients at least with early cancer, but the prognosis remains poor for those with advanced cancer.

New biomarkers of the disease that may contribute to establish the potential screening strategy would be of value for the early detection of individuals at high risk of the disease.

Nested case-control studies are often conducted to reduce the cost of cohort study dealing with such biomarkers, but yet produce the same findings with nearly the same level of precision.^6^ We used this approach to evaluate serum levels of insulin-like growth factor I, II, and binding protein 3 (IFG-I, IGF-II, and IGFBP-3), transforming growth factor *β*-1 (TGF *β*1), soluble fas (sFas) and superoxide dismutase activity (SOD) in stomach cancer cases diagnosed in the Japan Collaborative Cohort Study (JACC Study) for Evaluation of Cancer Risk sponsored by the Ministry of Education, Science, Sports and Culture of Japan (Monbusho) in relation to those levels in their controls in search of any novel markers associated with stomach cancer incidence.

Because *H. pylori* infection is causally related to stomach cancer, and may modify the levels of these serum makers, we evaluated the effect of *H. pylori* infection on these biomarkers as well.

## METHODS

### JACC Study

The study was part of the JACC Study, a nationwide multicenter collaborative study to prospectively evaluate various risk or protective factors on cancer mortality and incidence. The JACC study was started between 1988 and 1990, enrolling apparently healthy subjects living in 45 areas in Japan, collecting baseline data using a self-administered questionnaire. Sampling methods and detailed protocols of the JACC study are described elsewhere.^7^^-^^9^ We followed 110,792 subjects (46,465 men and 64,327 women), aged 40 to 79 years at baseline. About one-third of the cohort members (n = 39,293) also donated a residual serum sample (about 2 mL) used for the general health checkup. It was partitioned into 0.3 to 0.5 mL aliquots and stored at -80°C until laboratory analyses. Informed consent procedures were approved by the Ethics Committee of Medical Care and Research, University of Occupational and Environmental Health, Kitakyushu where the chief investigator of stomach cancer group is affiliated, and the Ethical Board of the Nagoya University School of Medicine, Japan where the present chairman of the JACC study is affiliated.

### Follow-up and Identification of Stomach Cancer Cases, and Selection of Control Subjects

Vital statuses of the participants were checked annually by each regional research center with permission to review their population-register sheets from the Ministry of Public Management, Home Affairs, Post and Telecommunications. Incidence of cancer was ascertained in 24 study areas (n = 65,184) and coded according to the tenth revision of International Classification of Diseases and the second edition of International Classification of Diseases for Oncology. These data were collected at the central office of the Research Committee.

We first restricted the subjects to those who lived in the study areas where cancer incidence was ascertained. We then excluded 857 participants with a self-reported history of cancer at any site. Among the remaining 64,327 subjects, diagnosis of stomach cancer 12 or more months after cohort recruitment was documented in 804 cases until the end of 1997. Serum had been obtained from 218 out of the initial 804 cases. However, 7 cases without enough serum for laboratory analysis, and one case without an eligible control subject were excluded. Thus, the study reported here included 210 cases (110 men and 100 women) for IGFs and SOD analysis, and 209 cases (109 men and 100 women) for sFas and TGF *β*1 analysis. Lag time between blood sampling and stomach cancer diagnosis varied between 12 and 113 months (median 50 months). Each of these subjects was matched with two control subjects for gender, age at recruitment (as near as possible) and study area, who had also provided an adequate baseline blood sample and who were alive as of the end of 1997. Owing to a lack of eligible subjects, a few sets contained only 1 control; thus, a total of 410 controls were available for IGFs and SOD analysis, and 409 for sFas and TGF *β*1 analysis. Because information on the location of cancer within the stomach or the histological type was not available in all cases, we did not use it to classify cases.

### Laboratory Assays

Serum samples from each case and matched controls were retrieved from storage and shipped on dry ice to the single laboratory (SRL, Inc., Hachioji, Japan) for the assay by trained staffs that were blinded to the case/control status of the samples. None of the samples had been previously defrosted.

Serum levels of IGF-I, II and IGFBP-3 were measured by immunoradiometric assay, using commercially available kits (Daiichi Radioisotope Lab., Tokyo). The mean intra-assay coefficient of variation on quality control serum samples was 2.15-3.53% for IGF-I, 2.74-4.45% for IGF-II and 3.16-4.19% for IGFBP-3. In a pilot study, we examined the stability of standard curves and the sensitivity and reproducibility of the assays. The range of reliable measurement for IGF-I was 40-2,050 ng/mL, 10-1,640 ng/mL for IGF-II, and 0.06-10.10 *μ*g/mL for IGFBP-3. The inter-assay coefficients of variation were 1.12-4.18%, 4.23-5.53%, and 5.28-8.89% for IGF-I, IGF-II and for IGFBP-3, respectively.

The total TGF *β*1 was measured by sandwich enzyme-linked immunosorbent assays (R&D Systems, Minneapolis, MN). Results were expressed in nanograms per milliliter (ng/mL). The assay range was 20-2,180 ng/mL. Intra- and inter-assay coefficients of variation were 2.67-6.79% and 4.17-6.16%, respectively. Serum SOD activity was estimated from the decreasing rate of nitrite produced by hydroxylamine and superoxide anions, based on an improved nitrite method.^10^ The assay range was 0.1-10.0 U/ml and the intra- and inter- assay coefficient of variation were 4.02-6.79% and 2.79-5.82%, respectively.

Soluble Fas was assayed by enzyme-linked immunosorbent assay using commercially available kits (MBL Co. Ltd., Tokyo). The assay range was 5.0-50.0 pg/mL, and the intra- and inter assay precision was 2.18-5.55% and 8.24-12.3%, respectively.

*H. pylori* infection was investigated serologically using HM-CAPTM (Enteric Products, Westbury, NY, USA) with antigen from Japanese (J-HM-CAP), and the serum titer of immunoglobulin G antibodies 2.3 or greater was defined as positive infection.

### Statistical Analysis

We compared baseline characteristics of case subjects and control subjects by one-way analysis of variance for continuous variables and *chi*-squared tests for categorical variables. Serum levels of SOD and sFas were not normally distributed (Kolmogorov-Smirnov test), thus natural logarithmically transformed in advance. We also compared the mean levels of these markers in subjects with *H. pylori* infection with those without an infection in the control group and case group separately. All reported p values are two-sided. All analyses were performed separately for men and women with SPSS^®^ statistical package for windows version 12.0.

## RESULTS

Table 1 shows the baseline characteristics of the 210 cases and the 410 matched controls. The average ages of male and female stomach cancer cases were 63.7 and 61.6 years, respectively. The proportion of individuals infected with *H. Pylori* was high even in control subjects (80.2% and 79.3% for men and women, respectively). However, it was higher in cases with stomach cancer among men and significantly higher among women (87.3% and 91.0% for men and women, respectively: P values for the chi-squared test were 0.12 in men, and 0.013 in women; case vs. control).

**Table 1.** Baseline characteristics of the study participants.

	Men (n=322)			Women (n=298)		
	
	Cases (*n*=110)	Controls (*n*=212)	P value*	Cases (*n*=100)	Controls (*n*=198)	P value*
Age (year)						
40-49	6 (5.5)	12 (5.7)	Matching factor	9 (9.0)	18 (9.1)	Matching factor
50-59	23 (20.9)	44 (20.8)		33 (33.0)	66 (33.3)	
60-69	54 (49.1)	108 (50.9)		40 (40.0)	79 (39.9)	
70-79	27 (24.5)	48 (22.6)		18 (18.0)	35 (17.0)	

Age (year): mean±SD	63.7±7.9	63.4±7.9		61.6±8.2	61.5±8.3	

*Helicobacter pylori* infection						
Present	96 (87.3)	170 (80.2 )	0.12	91 (91.0)	157 (79.3)	0.013
Absent	14 (12.7)	42 (19.8)		9 (9.0)	41 (20.7)	

* : P value by 2 × 2 *chi*-squared test.

SD : standard deviation.

Serum levels of IGF-I, II, and IGFBP-3, TGF *β*1, sFas, SOD in stomach cancer cases and their controls are presented in Table 2. Among 6 serum biomarkers tested for case-control differences, only sFas level in female stomach cancer cases were significantly higher than that of controls (2.22 pg/mL vs. 2.04 pg/mL, respectively; P = 0.013 by two-way ANOVA controlling for matching).

**Table 2.** Comparisons of serum levels of insulin-like growth factor I, II, and binding protein 3, transforming growth factor *β*-1, soluble fas ligand and superoxide dismutase activity in stomach cancer cases and their controls.

	Men (n=322)					Women (n=298)				
	
	Cases (*n*=110*)		Controls (*n*=110*)		P value^†^	Cases (*n*=100)		Controls (*n*=198)		P value^†^
	Mean	SD	Mean	SD		Mean	SD	Mean	SD	
IGF-I (ng/mL)	127	52	131	54	0.70	121	53	117	53	0.41
IFG-II (ng/mL)	548	127.4	571	139.2	0.13	618	122	607	118	0.40
IFGBP-3 (*μ*g/mL)	2.87	0.93	2.94	0.87	0.41	3.09	0.72	3.11	0.87	0.85
TGF *β*1 (ng/mL)	37.1	7.9	36.5	8.0	0.70	36.1	7.8	35.8	8.2	0.82
sFas^‡^ (pg/mL)	2.26	1.15	2.20	0.76	0.38	2.22	0.85	2.04	0.79	0.013
SOD^‡^ (U/mL)	2.83	1.67	2.82	1.44	0.84	3.09	1.62	3.03	1.49	0.68

IGF-I, insulin-like growth factor I; IGF-II, insulin-like growth factor II; IGFBP-3, insulin-like growth factor binding protein 3; TGF *β*1, transforming growth factor *β*-1; sFas, soluble fas ligand; SOD, superoxide dismutase activity.

* : n=109 and 211 for sFas and TGFb1 analyses

† : P value by two-way ANOVA controlling for matching variable.

‡ : Geometric mean and geometric standard deviations are presented.

SD: standard deviation.

Age-adjusted serum levels of IGF-I, II, and IGFBP-3, TGF *β*1, sFas, SOD in control subjects with and without *H. pylori* infection were compared in Table 3. In male controls, the mean value of serum IGF-I was higher in those without *H. pylori* infection than in those with the infection (150 ng/mL vs. 125 ng/mL, P = 0.011, respectively by one-way ANOVA). Serum sFas level in control subjects without *H. pylori* infection was slightly higher than in those with the infection in men (2.27 pg/mL vs. 2.21 pg/mL, P = 0.091).

**Table 3.** Comparisons of serum levels of insulin-like growth factor I, II, and binding protein 3, transforming growth factor *β*-1, soluble fas ligand and superoxide dismutase activity in the subjects with H. pylori infection and those without infection (within control participants).

	Men (n=212)					Women (n=198)				
	
	*H. pylor*i positive (*n*=170)		*H. pylori* negative (*n*=42)		P value*	*H. pylor*i positive (*n*=157)		*H. pylori* negative (*n*=41)		P value*
	Mean	SD	Mean	SD		Mean	SD	Mean	SD	
IGF-I (ng/mL)	125	51	150	57	0.011	118	53	124	53	0.48
IFG-II (ng/mL)	558	133	587	148	0.95	609	120	616	119	0.63
IFGBP-3 (*μ*g/mL)	2.85	0.84	3.22	1.04	0.18	3.09	0.81	3.20	0.87	0.41
TGF *β*1 (ng/mL)	36.9	7.9	35.7	8.0	0.20	36.1	8.1	35.0	7.6	0.30
sFas^†^ (pg/mL)	2.21	0.92	2.27	0.81	0.091	2.10	0.83	2.07	0.75	0.88
SOD^†^ (U/mL)	2.83	1.57	2.79	1.28	0.40	3.08	1.62	2.92	1.11	0.37

IGF-I, insulin-like growth factor I; IGF-II, insulin-like growth factor II; IGFBP-3, insulin-like growth factor binding protein 3; TGF *β*1, transforming growth factor *β*-1; sFas, soluble fas ligand; SOD, superoxide dismutase activity.

* : P value by one-way ANOVA controlling for age.

† : Geometric mean and geometric standard deviations are presented.

SD : standard deviation.

Age-adjusted serum levels of IGF-I, II, and IGFBP-3, TGF *β*1, sFas, SOD in case subjects with and without *H. pylori* infection were compared in Table 4. In female cases, the mean value of serum IGF-I was higher in those without *H. pylori* infection than in those with the infection (143 ng/mL vs. 119 ng/mL, P = 0.019, respectively by one-way ANOVA).

**Table 4.** Comparisons of serum levels of insulin-like growth factor I, II, and binding protein 3, transforming growth factor *β*-1, soluble fas ligand and superoxide dismutase activity in the subjects with H. pylori infection and those without infection (within case participants).

	Men (n=110)					Women (n=100)				
	
	*H. pylor*i positive (*n*=96)		*H. pylori* negative (*n*=14)		P value*	*H. pylor*i positive (*n*=91)		*H. pylori* negative (*n*=9)		P value*
	Mean	SD	Mean	SD		Mean	SD	Mean	SD	
IGF-I (ng/mL)	139	49	125	68	0.35	119	48	143	88	0.019
IFG-II (ng/mL)	545	124	569	152	0.53	615	120	644	150	0.29
IFGBP-3 (*μ*g/mL)	2.82	0.87	3.16	1.30	0.17	3.07	0.68	3.31	1.06	0.14
TGF[1 (ng/mL)	37.4	7.9	35.0	7.9	0.29	36.0	7.8	37.2	8.3	0.56
sFas^†^ (pg/mL)	2.23	1.18	2.26	1.15	0.90	2.27	0.81	2.21	0.86	0.56
SOD^†^ (U/mL)	2.96	1.54	2.82	1.70	0.72	3.15	2.02	3.09	1.59	0.88

IGF-I, insulin-like growth factor I; IGF-II, insulin-like growth factor II; IGFBP-3, insulin-like growth factor binding protein 3; TGF *β*1, transforming growth factor *β*-1; sFas, soluble fas ligand; SOD, superoxide dismutase activity.

* : P value by one-way ANOVA controlling for age.

† : Geometric mean and geometric standard deviations are presented.

SD : standard deviation.

## DISCUSSION

In this case-control study nested within the JACC study, the serum level of sFas was significantly higher in female stomach cancer cases, which indicated that sFas level is related to the increased risk of developing stomach cancer in women. It was also higher in male cancer cases but the difference was not statistically significant (P = 0.38).

The finding that the women who would eventually develop stomach cancer had a significantly higher level of serum sFas may be consistent with the previous report that showed the significantly higher serum sFas level in gastric adenocarcinoma patients, which was significantly reduced by gastrectomy.^11^ A statistically significant expression of tumoral Fas ligand with concomitant increment of serum sFas in gastric adenocarcinoma was also demonstrated, which would indicate the increase in the serum sFas level had been due to an existing tumor. In the present study, lag time between blood sampling and stomach cancer diagnosis varied between 12 and 113 months (median 50 months), and it is unclear whether latent tumor had already existed at the time of blood sampling, or other mechanisms exist that would increase serum sFas level which initiate or promote tumorigenesis.

Serum sFas level was slightly higher in individuals without *H. pylori* infection than in those with the infection of borderline significance (P = 0.091) in male controls in the present study. The serum sFas level did not differ between with or without *H. pylori* infection in women. One study that examined the response of *H. pylori* eradication in gastric MALT lymphoma showed that the decrease in the serum sFas level would be a useful marker of successful treatment.^12^ The other study showed that *H. pylori* caused a selective up-regulation of Fas mRNA and the protein expression in gastric cancer cells.^13^ The mean serum sFas level in male case subjects with *H. pylori* infection did not differ from those in subjects without the infection (2.26 pg/mL vs. 2.23 pg/mL, P = 0.90). No conclusion could be drawn from the present study about the relationship between sFas serum level ant *H. pylori* infection, which warrants further investigation.

Although a previous study revealed that serum levels of Cu/Zn SOD activity were significantly elevated in gastric cancer patients compared with apparently healthy controls,^14^ we did not find any significant association in the present study.

The significant associations between serum IGF-I, II, or IGFBP-3 and stomach cancer have been observed in the previous studies dealing with disease patients and their controls.^15^^,^^16^ We did not, however, find any significant differences in IGFs levels between cases and controls, suggesting the alterations observed in the previous studies might be due to existing tumors. Because this is the first study that examined the difference in serum IGFs in a prospective study, further study that tests our finding is needed.

Serum IGF-I was significantly higher in male control subjects without *H. pylori* infection than in men (control) with the infection in the present study. Serum IGF-I was significantly higher in female case subjects without *H. pylori* infection than in women (case) with the infection in the present study. One previous study did not find any difference in serum IGF-I level according to *H. pylori* infection status.^17^ Further study with more precise diagnosis of *H. pylori* infection in the gastric mucosa may be needed.

We found no association between TGF *β*1 levels and stomach cancer. In previous clinical studies, elevated serum TGF *β*1 levels have been observed in gastric cancer patients with poor prognoses,^18^ those in an advanced stage, or those with poorly differentiated or invasive type adenocarcinoma.^19^ Others, however, did not necessarily find such an association of blood TGF *β*1 levels with tumor stages.^20^ Elevated TGF *β*1 serum levels observed in certain gastric cancer patients may be due to an existing tumor.

In conclusion, none of the biomarkers consistently predicted future risk of stomach cancer in both men and women in the present analysis. Serum sFas level in women, however, should be studied much more thoroughly whether it provides meaningful refinement of risk stratification, or it elucidate the mechanisms of tumorigenesis in women.

## MEMBER LIST OF THE JACC STUDY GROUP

The present investigators involved, with the co-authorship of this paper, in the JACC Study and their affiliations are as follows: Dr. Akiko Tamakoshi (present chairman of the study group), Nagoya University Graduate School of Medicine; Dr. Mitsuru Mori, Sapporo Medical University School of Medicine; Dr. Yutaka Motohashi, Akita University School of Medicine; Dr. Ichiro Tsuji, Tohoku University Graduate School of Medicine; Dr. Yosikazu Nakamura, Jichi Medical School; Dr. Hiroyasu Iso, Institute of Community Medicine, University of Tsukuba; Dr. Haruo Mikami, Chiba Cancer Center; Dr. Yutaka Inaba, Juntendo University School of Medicine; Dr. Yoshiharu Hoshiyama, University of Human Arts and Sciences; Dr. Hiroshi Suzuki, Niigata University School of Medicine; Dr. Hiroyuki Shimizu, Gifu University School of Medicine; Dr. Hideaki Toyoshima, Nagoya University Graduate School of Medicine; Dr. Kenji Wakai, Aichi Cancer Center Research Institute; Dr. Shinkan Tokudome, Nagoya City University Graduate School of Medical Sciences; Dr. Yoshinori Ito, Fujita Health University School of Health Sciences; Dr. Shuji Hashimoto, Fujita Health University School of Medicine; Dr. Shogo Kikuchi, Aichi Medical University School of Medicine; Dr. Akio Koizumi, Graduate School of Medicine and Faculty of Medicine, Kyoto University; Dr. Takashi Kawamura, Kyoto University Center for Student Health; Dr. Yoshiyuki Watanabe, Kyoto Prefectural University of Medicine Graduate School of Medical Science; Dr. Tsuneharu Miki, Graduate School of Medical Science, Kyoto Prefectural University of Medicine; Dr. Chigusa Date, Faculty of Human Environmental Sciences, Mukogawa Women’s University ; Dr. Kiyomi Sakata, Wakayama Medical University; Dr. Takayuki Nose, Tottori University Faculty of Medicine; Dr. Norihiko Hayakawa, Research Institute for Radiation Biology and Medicine, Hiroshima University; Dr. Takesumi Yoshimura, Fukuoka Institute of Health and Environmental Sciences; Dr. Akira Shibata, Kurume University School of Medicine; Dr. Naoyuki Okamoto, Kanagawa Cancer Center; Dr. Hideo Shio, Moriyama Municipal Hospital; Dr. Yoshiyuki Ohno, Asahi Rosai Hospital; Dr. Tomoyuki Kitagawa, Cancer Institute of the Japanese Foundation for Cancer Research; Dr. Toshio Kuroki, Gifu University; and Dr. Kazuo Tajima, Aichi Cancer Center Research Institute.
